# Evaluating measurement uncertainty in Brillouin distributed optical fibre sensors using image denoising

**DOI:** 10.1038/s41467-021-25114-4

**Published:** 2021-08-12

**Authors:** Marcelo A. Soto, Zhisheng Yang, Jaime A. Ramírez, Simon Zaslawski, Luc Thévenaz

**Affiliations:** 1 EPFL Swiss Federal Institute of Technology, Institute of Electrical Engineering, SCI STI LT, Station 11, Lausanne, Switzerland; 2 Department of Electronics Engineering, Universidad Técnica Federico Santa María, Valparaíso, Chile; 3 State Key Laboratory of Information Photonics & Optical Communications, Beijing University of Posts and Telecommunications, Beijing, China

**Keywords:** Electrical and electronic engineering, Fibre optics and optical communications, Optical sensors

**arising from** M. A. Soto et al. *Nature Communications* 10.1038/ncomms10870 (2016)

In 2016, our research team proposed in an issue of *Nature Communications*^1^ the use of multidimensional signal processing, especially image denoising techniques, to improve the signal-to-noise ratio (SNR) of distributed optical fibre sensors. The benefits of the method were demonstrated for distributed Raman and Brillouin sensors, both proving a significant performance improvement. Here we show that, while the SNR enhancement for the case of Brillouin distributed sensing was correctly estimated in our publication^1^, an overestimation of the Brillouin frequency uncertainty reduction was reported as a result of the inadvertent use of a conventional methodology for performance evaluation. Based on a better understanding of the impact of image denoising applied to Brillouin distributed sensors, here we report a revised estimation of the Brillouin-frequency shift (BFS) uncertainty obtained after image denoising, verifying that although 2D image denoising can significantly improve the measurement SNR, this cannot be fully transferred to the overall Brillouin sensing performance.

Based on novel findings and a deeper understanding of the method^2^, and for the sake of clarity, we start by clearly stating the fundamental limitations and real benefits of image denoising applied to Brillouin distributed sensing, by partially reusing the analysis and conclusions drawn in ref. ^2^. In the following, the effects of 2D filtering in the 2 dimensions of the matrix containing the measured data (i.e. in the Brillouin-spatial domain along the fibre and in the Brillouin-frequency domain describing each local Brillouin gain spectrum) are separately addressed.

## Effects of 2D filtering in the Brillouin-spatial domain

Traditional noise reduction filters correspond to lowpass filters that can efficiently improve the quality of a signal by eliminating noise out of the signal spectral band. When applied to time-domain traces of a distributed fibre sensor, the filter has a longitudinal smoothing effect that results from the convolution between the filter impulse response and the noisy input time-domain trace. At the filter output, adjacent signal samples increase their degree of mutual correlation, which in the context of distributed sensing could lead to a reduction of the spatial resolution of the sensor. The proper design of a denoising strategy requires that the filter reduces the noise bandwidth without impacting the useful signal bandwidth, so as to avoid degrading the spatial resolution of the sensor.

Such a universal strategy also applies to 2D denoising methods presented in ref. ^1^, i.e., nonlocal means (NLM)^3^ and wavelet denoising (WD)^4^, requiring to carefully define their filter parameters to match the noise bandwidth to the relevant Brillouin signal bandwidth, which is determined by the spatial resolution of the sensor, even though the adaptive feature of these techniques looks appealing at first glance. This implies that the spatial-domain filtering contribution of 2D denoising is only beneficial when the noise bandwidth in the electrical domain is larger than the useful signal bandwidth. In other words, if the electrical bandwidth has been optimised to match the signal bandwidth, there will be no real benefit provided by the spatial-domain filtering component of the 2D denoising^2^. In addition, the sampling rate of the Brillouin-spatial-domain traces must fulfil the Nyquist criterion for the noise bandwidth to avoid aliasing of the noise^2^.

## Effects of 2D filtering in the Brillouin-frequency domain

Image denoising can also correlate adjacent data points describing the Brillouin gain spectrum at each fibre position. This effect can also contribute to the SNR improvement of the measurements. Recent findings have however demonstrated that the attained SNR improvement cannot be straightforwardly translated into a BFS uncertainty improvement^2^. The reason lies in the following feature: the noise removal contribution from the 2D filter in the spectral domain turns out to be redundant with the spectral fitting used to determine the Brillouin gain peak frequency^2^; and therefore, the filtering in the spectral domain should have no impact on improving the BFS uncertainty, even if a large SNR enhancement can be verified on BOTDA traces.

In awareness of these fundamental limitations, the methodology to evaluate the sensor performance is revisited hereafter, aiming at correcting the overestimation of the BFS accuracy in our previous publication^1^.

## Estimating the BFS uncertainty improvement—revisited

The estimation of the SNR and BFS uncertainty of a standard Brillouin sensor normally assumes that calculations of the statistical values in the sequential domain (i.e. using successive measurements) and in the distance domain (i.e. along a uniform fibre segment using a single acquisition) are equivalent in the context of a purely additive white Gaussian noise. This feature has been verified several times by the scientific community and its validity is commonly applicable to many practical scenarios. This assumption is however only valid if specific conditions are satisfied in both sequential and distance domain. Under such conditions, the following two approaches can be followed:

1. In the *distance-domain approach*, a spatial moving window is used to calculate the mean value and standard deviation over the temporal trace acquired at maximum Brillouin gain condition (for SNR estimation), as well as to calculate the standard deviation of the BFS distribution (for the frequency error estimation). The statistical values are obtained along the fibre from a single measurement using a proper spatial window length. To avoid including any longitudinal BFS variation that can bias the frequency error estimation, a reference BFS trace (‘ideally noiseless’, i.e. acquired with a large number of averages) must be subtracted from the actual measured BFS distribution before calculating the longitudinal BFS standard deviation.
2. In the *sequential-domain approach,* several consecutive measurements are used to calculate the statistical values at each fibre position. The sequence length must be chosen to minimise any temporal drifts of the environmental quantities, such as temperature and strain.

In a well-designed conventional Brillouin sensor, noise affecting the Brillouin measurements can be safely considered as additive white Gaussian noise, especially over long sensing ranges^5^. Under such a situation it turns out to be equivalent to estimate the measurement SNR and BFS uncertainty in either sequential or distance domains (provided that the estimation does not contain longitudinal and temporal BFS fluctuations). Based on this property, the data processing used to obtain results in Fig. 3 of ref. ^1^, showing the SNR and frequency uncertainties of a Brillouin optical time-domain analyser (BOTDA), followed the distance-domain approach. In the case of estimating the frequency uncertainty of the raw and denoised data, a reference BFS trace was subtracted from the actual BFS trace to get rid of longitudinal Brillouin-frequency variations before calculating longitudinal standard deviations.

In the following, we show that the BFS uncertainty reported in ref. ^1^ (obtained with the distance-domain approach) resulted to be unwittingly overestimated when compared to the sequential-domain approach. To perform this comparison, the following analysis shows some results obtained after processing a series of BOTDA measurements acquired under similar conditions than the ones reported in ref. ^1^. 2D filtering using nonlocal means (NLM)^3^ and wavelet denoising (WD)^4^ has been applied to measured data using the same algorithms and parameters previously used^1^. The SNR and frequency uncertainty are then calculated in both sequential and distance domains for comparison.

**Fig. 1:**
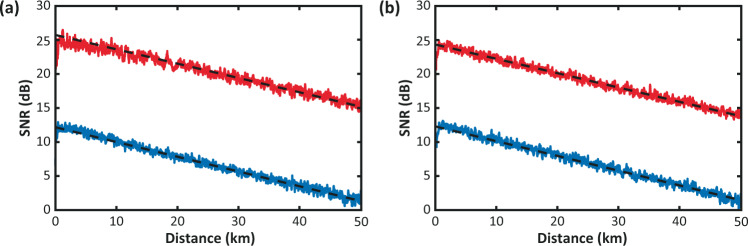
Impact of NLM denoising on estimated SNR. SNR profile versus distance for raw BOTDA data (blue) and NLM-filtered data (red), using statistical values estimated in **a** distance domain, and **b** sequential domain.

**Fig. 2:**
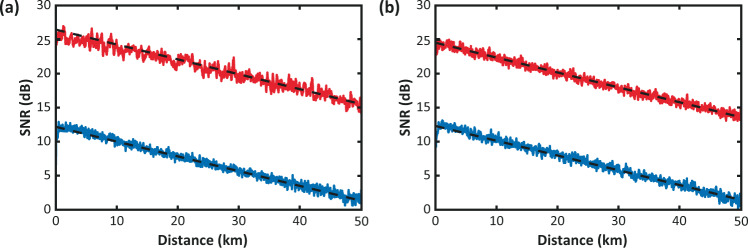
Impact of wavelet denoising on estimated SNR. SNR profile versus distance for raw BOTDA data (blue) and WD-filtered data (red), using statistical values estimated in **a** distance domain, and **b** sequential domain.

Figures 1 and 2 show a comparison of the SNR obtained following (a) the distance-domain approach, and (b) the sequential-domain approach, for the NLM and WD cases, respectively. Results point out that the SNR estimations using the raw BOTDA data in both distance and sequential domains are very similar to each other. With the use of 2D image denoising, the raw SNR of 1.6 dB at 50 km distance is improved up to 15.0 and 15.5 dB, for the NLM and WD cases, when estimated in the distance domain, well agreeing with the previous results^1^. The sequential-domain approach is also consistent with previous results, although showing slightly lower SNR values after 2D filtering, thus resulting in 13.8 and 13.5 dB SNR at a 50 km distance for each of the respective cases.

**Fig. 3:**
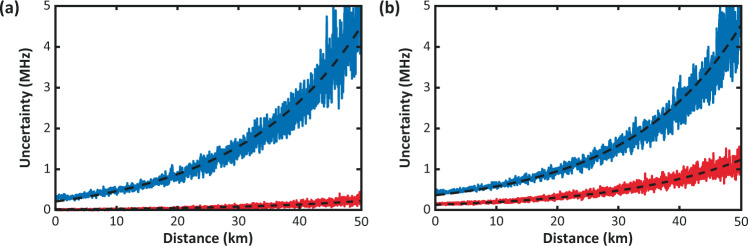
Impact of NLM denoising on estimated BFS uncertainty. Frequency uncertainty versus distance for raw BOTDA data (blue) and NLM-filtered data (red), using statistical values estimated in **a** the distance domain, and **b** the sequential domain.

**Fig. 4:**
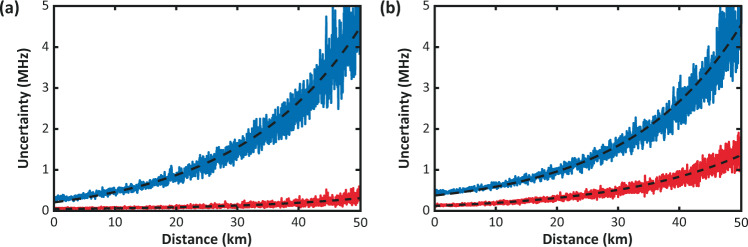
Impact of wavelet denoising on estimated BFS uncertainty. Frequency uncertainty versus distance for raw BOTDA data (blue) and WD-filtered data (red), using statistical values estimated in **a** the distance domain, and **b** the sequential domain.

Interesting results are shown in Figs. 3 and 4, which report comparisons of the BFS uncertainties obtained following (a) the distance-domain approach, and (b) the sequential-domain approach, for the NLM and WD cases, respectively. The Brillouin-frequency errors obtained in distance and sequential domains for the case of raw data are similar to each other, as usually occurs in conventional Brillouin distributed sensors. In addition, results obtained following the distance-domain approach are equivalent to the ones reported in ref. ^1^, showing uncertainties of 0.22 and 0.23 MHz at 50 km distance for the NLM and WD cases, respectively. However, significant differences can be observed when the sequential-domain approach is applied. In this case, the BFS uncertainty is improved from 4.5 MHz (raw data) down to 1.2 and 1.3 MHz, for the NLM and WD methods respectively; thus, representing a much smaller BFS uncertainty improvement when compared to the estimations obtained in the distance domain.

Note that the improvement from 4.5 MHz down to 0.2 MHz obtained in the distance-domain approach closely matches (although not precisely) the expected BFS enhancement that would have been obtained from the entire SNR enhancement^6^. This kind of result led us to assume that the BFS estimations obtained following the distance domain approach in ref. ^1^ were correct and closely matching the expected theoretical BFS enhancement. Based on a better understanding of the impact of image denoising on the Brillouin sensing data, now it becomes clear that the actual BFS improvement attained by the method is only provided by the filtering effect in distance domain of BOTDA traces and not by the contribution of the filter in the Brillouin-frequency domain. The correct estimation of the BFS uncertainty is given by the sequential-domain approach and the additional BFS enhancement obtained in the distance-domain approach is only fictitious and does not represent a correct evaluation of the uncertainty because of the added correlation between adjacent data points caused by the photodetection bandwidth and the 2D image denoising filters. These points turn out to be no longer fully statistically independent^6^.

In conclusion, special attention must be paid when calculating the BFS uncertainty of Brillouin distributed sensors when using image denoising. The correlation induced on closely located data points makes the distance-domain approach an unreliable approach to estimate the performance of the sensor when using image denoising. In contrast, the sequential-domain approach turns out to be reliable to estimate the measurement uncertainty and secures a full statistical independence. Based on the understanding of this specific behaviour occurring only when image denoising is applied to Brillouin distributed sensors, a corrected evaluation of the BFS uncertainty for the data reported in ref. ^1^, has been here presented, which agrees well with the theory and results reported in ref. ^2^.

## Data Availability

The data that support the findings of this study are available from the corresponding author upon reasonable request.
